# Demonstrative Midwifery

**Published:** 1850-06

**Authors:** 


### THE WESTERN JOURNAL.

THIRD SERIES.—VOL. V.—NO. VI.

LOUISVILLE, JUNE 1, 1850.

### DEMONSTRATIVE MIDWIFERY.

We are gratified in seeing that the Medical Journals, of this country are expressing themselves very properly upon the recent *emeute* got up in Buffalo, by some seventeen physicians, on the subject of demonstrativ midwifery. In addition to the remarks made in the May number oı this Journal, very excellent and appropriate editorials have appeared in the New York Journal of Medicine, the New Orleans Medical Journal, and the Western Lancet, published at Cincinnati. They all speak approvingly of the course of the Professor of Obstetrics, in the Buffalo Medical School.

It is a matter of surprise and sorrow to us, that even seventeen medical men in one city, could be induced to sign such a letter as the one addressed to Prof. Austin Flint, and to the character of which we referred in our last number. We can easily imagine precisely such professional *esprit du corps*, and enterprise, as were exhibited by the immortal seventeen at Buffalo, manifesting itself in Rome, against Galen's desires for anatomical knowledge. A letter could easily have obtained numerous signatures, denouncing *post mortem* examinations, and dissections of the human body, as "unprofessional in manner, grossly offensive, alike to morality and common decency," subversive of the public virtue, and an outrage upon the gods. There is but little doubt, that just such exemplars of professional honor, advancement, and utility, as are to be found in Buffalo, were among those great names in the medicine of Rome, who drove Galen away from the city, and kept him down, until the fires of his genius consumed the moths that flitted around the flames of the reputation of the illustrious Greek.

Dr. Wm. Shippen had to stem the tide of just such absurd and groundless prejudices, as the Buffalo medical protestants urge, when he attempted to *practice* midwifery in Philadelphia. A great outcry was raised against the illustrious Shippen—his attempt was looked upon as "unprofessional in manner, and grossly offensive to morality and common decency," but Shippen survived these "ghostly rappings," and paved the way to the very general employment of male accoucheurs. By the way, would it not be well enough for the seventeen Buffalo gentlemen to consult the Rochester rapping folks, on what is "unprofessional in manner, and grossly offensive alike to morality and common decency?" Our Buffalo indignants should remember too, how nearly Cotton Mather and Boylston came to the loss of their lives by introducing inoculation. The "inflammatory" doctors of the day appealed to the "masses" and raised a storm.

The various medical journals, we have named above, take strong ground in favor of the utility of demonstrative midwifery. In connection with that point we have no kind of hesitation in saying, that there is no department of medical teaching, in which demonstrations—actual clinics, are more important. The eloquence of Buffon could not pourtray by description, to an adult, anything like as correct an idea of an elephant, as a child would acquire from a few moments' view of the living animal; nor could all the teaching of Cazeaux or Churchill convey to students such ideas of the mechanism of labor, as they would derive from attendance upon one case. We have been often requested by students of the medical school, of this city, for permission to attend upon some case of obstetrics with us, and opportunities have occasionally occurred for granting the privilege. Some of these students were young men who had graduated with great honor, and who could describe all of Baudalocque's presentations with the most perfect accuracy, but when they examined for the particular presentation in the living case, they were unable to tell whether they were about to encounter a hip, shoulder, or head presentation. And these gentlemen have all declared that they derived more useful knowledge from the actualities of a labor, than from all their previous instruction. Mannikin instruction is but the shadowy form of an obstetrical clinic; the living case is absolutely essential, to the proper preparation of a graduate, for passing into practice, and all efforts for paving the way towards an extended series of demonstrative midwifery, should be hailed by the profession, and by the public as laudable, and worthy of all honor and praise. The law is fierce upon forced resources for dissection, but neither the law, nor public sentiment attempts to disturb those voluntary gifts that are occasionally bestowed upon the anatomist. And this marks the line for demonstrative midwifery. If females can be found, who are willing to make themselves a part of a successful course of obstetrical instruction, they should be encouraged in the good work, and those *medical* men, who attempt to obstruct such progress, and who, with incendiary motives, talk of getting up mass meetings on the subject, deserve to be branded as unworthy members of a noble profession, and as recreants to the cause of science. They are "unprofessional in manner and grossly offensive, alike to morality and common decency." It is the duty of every medical man to advance his profession by every means in his power, and all the resources of teaching will never boast of preparing neophytes, too well for practice.

On the subject of the morality of demonstrative midwifery, we expressed our views in the May nnmber of this Journal, and we are convinced that demonstrative midwifery may be so conducted as to be of vast utility to students, and be quite as moral as a lecture upon chemistry. Those females who may be induced to submit themselves for demonstration are not necessarily immoral, there is no kind of immorality in the teacher's desire to thoroughly qualify his students, and there can be none in the aspirations of the students after knowledge that is of the highest importance to them. Where then is the possibility of wrong to any one, or to anything in this matter? We hope to see the day when demonstrative midwifery will be an essential part of medical teaching, and that day will assuredly come, as certainly as other clinical teaching has come to be regarded as necessary. The necessity now felt, every where for hospital instruction—a necessity that is driving medical schools to large cities, will grow until demonstrative midwifery is placed upon a secure basis.

The prudish "Miss Nancies," of Buffalo, have, unintentionally, conferred a benefit on the medical profession. Their excessive modesty and shame-facedness have aroused attention to the subject of clinical midwifery, and this attention will urge forward the good work. We can easily imagine an innocent, childlike simplicity, that would put pantalets upon the legs of a piano, and that would screen with a veil, everything capable of exciting prurient ideas, but we do not like to see this excessive flirtation with modesty, introduced into medical teaching. A misplaced irritation of the blushing organs operated upon the seventeen Buffalo doctors, and induced them to ease their tender consciences, by a protest. May the Heavens smile serenely over their innocent slumbers! But while they sleep with Rip Van Winkle devotion, or with Barney O'Rierdon's "attention," we pray them not to disturb medical teaching with their snoring. They may sleep as much as they please, but the profession should be awake. The oil in the lamps of the "seventeen" foolish virgins may burn out, but we wish to see the lights of the profession continually replenished, so that perpetual lamps may be lighting the path of progression.

